# Profanity

**Published:** 1876-08

**Authors:** 


We always admired the course pursued by a mother, who whenever she heard her little son make use of a profane or vulgar word, would at once thoroughly wash out his mouth with soap and water. We can hardly walk the length of one block in any street of any city—and it is far worse in country villages—without hearing the most shocking oaths from the mouths of men whose appearance indicate respectability. It is a wonder that such men will take the trouble to degrade themselves in the estimation of all decent people. It is not to be supposed that these people intend any irreverence to the Creator. It is simply a habit that many really kind hearted and honorable men thoughtlessly fall into. Could they "see themselves as others see" them, could they realize how it lowers them in the estimation of all whose good opinion they desire, profanity would be confined to a class of creatures so low in the scale of decency, that oaths in comparison to their other vices are adornments.

